# Mouse Models as a Translational Platform for the Development of New Therapeutic Agents in Multiple Myeloma

**DOI:** 10.2174/156800912802429292

**Published:** 2012-09

**Authors:** P Tassone, P Neri, R Burger, MT Di Martino, E Leone, N Amodio, M Caraglia, P Tagliaferri

**Affiliations:** 1 Medical Oncology, Department of Experimental and Clinical Medicine, Magna Græcia University and Tommaso Campanella Cancer Center, Catanzaro 88100, Italy; 2 Temple University’s College of Science & Technology, Philadelphia, PA, USA; 3 Division of Hematology and Bone Marrow Transplant, University of Calgary, Calgary, AB, Canada; 4 Division of Stem Cell Transplantation and Immunotherapy, 2^nd^ Department of Medicine, University of Kiel, 24105 Kiel, Germany; 5 Department of Biochemistry and Biophysics, Second University of Naples, Naples, Italy

**Keywords:** Microenvironment, mouse models, multiple myeloma, scaffolds, SCID-hu, SCID-rab, SCID-synth-hu, 5TMM.

## Abstract

Mouse models of multiple myeloma (MM) are basic tools for translational research and play a fundamental role in the development of new therapeutics against plasma cell malignancies. All available models, including transplantable murine tumors in syngenic mice, xenografts of established human cell lines in immunocompromised mice and transgenic models that mirror specific steps of MM pathogenesis, have demonstrated some weaknesses in predicting clinical results, particularly for new drugs targeting the human bone marrow microenvironment (huBMM). The recent interest to models recapitulating the *in vivo* growth of primary MM cells in a human (SCID-hu) or humanized (SCID-synth-hu) host recipient has provided powerful platforms for the investigation of new compounds targeting MM and/or its huBMM. Here, we review and discuss strengths and weaknesses of the key *in vivo* models that are currently utilized in the MM preclinical investigation.

## INTRODUCTION

Multiple myeloma (MM) is a plasma cell malignancy in which tumor cells accumulate into the bone marrow (BM) and produce abnormal amounts of a monoclonal protein. Despite all recent achievements in the understanding of the pathophysiology of the disease and the availability of new therapeutics, MM remains incurable and runs a progressive and lethal course [1-5]. One of the most important mechanisms involved in the development of MM drug resistance is the block of drug-induced cell death via activation of autocrine or paracrine signaling loops in the context of the human bone marrow microenvironment (huBMM) [6-9]. It appears clear now that the huBMM plays a crucial role in MM cell growth and expansion as well as in tumour cell survival and drug resistance and therefore by itself is one of the most interesting potential targets in MM treatment [6, 7, 10-12]. However, the lack of experimental models recapitulating the natural *milieu*, where the disease takes place, has been the major limitation in the study of the interaction between MM cells and its microenvironment. Most of presently available *in vivo* models of murine or human MM in fact do not reproduce the huBMM and therefore are not suitable systems to study mechanisms and activity of new agents targeting both tumor cells and its natural environment. However, some *in vivo* murine models (SCID-hu, SCID-*synth-hu*) provide reliable preclinical tools for pathophysiological and therapeutical studies with agents targeting MM cells within their specific huBMM. Herein, we discuss on the strengths and weaknesses of the most widely used *in vivo* experimental systems for MM. 

## THE MURINE 5T MODEL 

The 5TMM is an *in vivo* model of murine disease based on the serial transplantation of syngenic recipients with murine MM cells that spontaneously arose in C57BL/KaLwRij aging mice [13, 14]. So far, different 5TMM lines have been established with the 5T2MM and 5T33MM being the more commonly used mouse models. Compared to the human disease, they share some common key features such as the development of monoclonal gammopathy, the renal involvement and the hypercalcemia. Mice bearing MM present infiltration of the BM and show bone lesions [15] characterized by increased osteoclast activity [16]. Among the 5T series, the 5T2MM model represents the most similar to the human disease, while the 5T33MM model shows aggressive features similar to advanced MM with spread of tumor cells to the spleen and liver [17]. In the past, these models have considerably contributed to the understanding of the MM pathophysiology [18, 19] including the role of osteoprotegerin and RANK/RANKL interaction [20], anti-tumor activity of bisphosphonates [21] and other novel agents [22-26]. However, while the advantage of 5T models is the reproduction of a murine disease within syngenic and immunocompetent mice, the major limitation is that the murine MM presents obvious differences in its biology as compared to human disease. In addition, the murine MM cell lines used for serial transplantation do not recapitulate the wide spectrum of genetic heterogeneity, which indeed characterizes the human disease, nor these models allow the study of MM interaction with the huBMM. 

## GENETICALLY ENGINEERED MOUSE MODELS

Several strategies have been used to develop genetically engineered models of malignancies [27-30]. In general, DNA constructs generated by coupling gene sequences with specific or universal promoters and/or enhancers are microinjected into fertilized 1-2 day old mouse eggs that are then surgically implanted into surrogate mothers. In MM a major effort has been directed towards the construction of mice carrying mutated MM relevant oncogenes and tumor suppressor genes expressed in the B-cell lineage. 

Engineered mouse models of human plasma cell neoplasms are based on the transgenic expression in B-cells of IL-6 [31], the NPM-ALK fusion protein [32] and deregulation of c-Myc and Bcl-xl [33, 34]. C57Bl6/J mice with activated c-myc in post-germinal B-cells (Vk*myc) spontaneously develop monoclonal gammopathies and plasma cell expansion in the BM but not in secondary lymphoid organs. They exhibit monoclonal paraproteinemia, anemia and decreased bone mineral density. Interestingly, these mice are also responsive to drugs commonly used for therapy and are predictive of clinical efficacy [35, 36].

In the pEμXBP-1 transgenic model, the spliced form of XBP-1 (XBP-1s) is targeted to the B cell lineage under the control of the IgH promoter and enhancer elements (pEμ) [37]. XBP-1 is a transcription factor that is required for plasma cell differentiation and is overexpressed in MM cells *versus* normal plasma cells [38]. The pEμXBP-1 transgenic mice develop features closely resembling MGUS and a significant proportion progresses to MM by one year of age. They have marked elevation of serum IgM and IgG, expansion of the plasma cell population in the BM (with plasma cells ranging from 5 to 30% of total BM cellularity), development of plasmacytic tumors resembling human MGUS or MM and eventually presence of lytic bone lesions. The long latency period in the development of MM indicates that additional genetic lesions are required for full malignant transformation. In addition, the transcriptional profiles of the lymphoid and MM cells show deregulation of genes with known dysfunction in human MM including cyclin D1, IL-6 receptor (gp80), gp130, MAF, MAFB, BAFF and APRIL. Overall, the pEμXBP-1 transgenic model is particularly suitable to study the role of genetic lesions in the evolution and progression of the disease. 

A new transgenic mouse model that specifically expresses c-MAF in B-cells has been recently reported [39]. These mice show expansion of plasma cells, hyperglobulinemia and high expression of c-MAF target genes, CCND2 and ITGB7*, *thus providing a good model of human MM carrying the t(14;16)(q32;q23) chromosomal translocation. 

In general, transgenic mice can be useful for studying the role of oncogenes for normal cell differentiation and malignant transformation. They are also very useful for testing new agents against proteins that are generated by specific genetic alterations. However, in some models of gene knock-in or knock-out mice, the tumor does not arise in a natural environment. Instead, the tumor develops in a mutated murine micro-environment which resembles the inherited cancer onset scenario. The use of more advanced engineered models for the screening of investigational agents is limited by the obvious differences between the murine and the human stroma, the impossibility to reproduce different tumor stages or some difficulties to follow the tumor development. Finally, transgenic mouse strains are labor intensive, time-consuming and can be very costly, therefore limiting their use by many institutions.

## CONVENTIONAL SUBCUTANEOUS OR DIFFUSE XENOGRAFTS OF HUMAN MM CELL LINES 

Subcutaneous or diffuse xenograft models are based on local or systemic injection of established human MM cell lines into immune compromised mice (e.g. nude, SCID, SCID/beige, NOD/SCID, NOD/SCID/γc null) to produce local or disseminated disease, respectively. These tumors generally grow quickly and in most of the cases in a non-orthotopic environment such as the skin, liver, lung, or other organs. In some cases the murine BMM can be the initial homing site of the disease which continues its course involving additional organs [40-42]. Subcutaneous tumors can be easily monitored by measuring the mass size and its changes by a simple caliper. Changes in the tumor volume represent the most common primary endpoint of drug activity in these models [43-46]. Another important endpoint commonly used in such models is the mouse survival, defined by the time interval between the start of the experiment and either spontaneous mouse death or sacrifice. A variety of innovative drugs have been evaluated by the use of such models including immunoconjugates, proteasome inhibitors, immunomodulatory drugs, and many others [43-56]. On the other hand, diffuse xenograft models of human MM are difficult with regard the monitoring of disease localization, spatio-temporal progression and total burden of the disease due to various sites of organ involvement not easily accessible for real-time monitoring. Even if computerized tomography (CT) and magnetic resonance imaging (MRI) have proven suitable to circumvent these limitations, the cost of these technological resources is still prohibitive for many institutions. The difficulty of monitoring disease localization and progression can be partially overcome by the use of human MM cell lines stably expressing constructs for markers detectable by whole-body bioluminescence and/or fluorescence imaging, which allows the external visualization of tumor masses [57]. However, some intrinsic weaknesses remain regarding the use of both subcutaneous and diffuse models for the preclinical evaluation of anti-MM agents. These models are usually based on the use of human plasma cell lines which are often established with primary cells obtained from extramedullary disease of advanced MM patients (peripheral blood or pleural effusion of patients with plasma cell leukemia), and therefore are not fully consistent with the biological behaviour of intramedullary disease in which MM cells closely interact and depend from the huBBM [9, 58]. Moreover, cell lines *i*) are unstable over time and acquire additional genetic abnormalities conferring an even more dedifferentiated and aggressive phenotype different from the original disease [58, 59]; *ii*) lack the heterogeneity observed in MM patients; *iii*) show a higher S-phase fraction of proliferating cells compared to primary MM cells; *iv*) sometimes are EBV positive representing a B-lymphoproliferative and not a plasma cell phenotype [58], and *v*) produce poorly vascularized xenografts that grow rapidly within few weeks and present wide necrotic areas. Interestingly, among the most used MM cell lines, there is virtually none representing the hyperdiploid D1 group, which is likely an early-phase disease strictly dependent on the BMM [58]. A further key point to be considered is that in these models MM cell lines grow in a non-human and non-orthotopic environment. Even diffuse xenografts with initial localization in the mouse BM quickly disseminate to extramedullary sites. This is an important key limitation of these systems since they partially or completely lack the intrinsic dependency of MM cells from huBMM, therefore reducing the clinical translational value of these models. Overall, while these models are suitable for the study of cytotoxic agents or for a quick screening of investigational drugs, they do not provide a biological scenario similar to human disease and therefore cannot provide insights into mechanisms of novels drugs targeting the huBMM *milieu*.

## THE SCID-hu MOUSE MODEL

The engraftment of primary MM cells in immuno-compromised mice may be achievable, but occurs in the peritoneal cavity and lymph nodes and not in the murine bone marrow[56] or with cells from advanced disease only [57, 58, 61, 62]. The idea to allow primary MM cells, freshly explanted from bone marrow aspirates of non-advanced patients, to engraft within a huBMM, has been realized with the introduction of the SCID-hu mouse model. The implantation of intact human hemato-lymphoid organs (thymus/liver and bone) into the SCID mouse was initially developed to study the human hematopoiesis [63] and the pathophysiology of HIV induced disease. The model was also used for evaluation of cytokines [64] and gene therapies [65] and for studying the growth of primary leukemia and lymphoma cells [66-68]. Urashima *et al*. [69] were the first to show engraftment and proliferation of human MM cell lines in human fetal bone chips implanted into irradiated SCID mice. By histologic examinations they have also demonstrated the ability of MM cells to home exclusively to the human but not the murine BM. Subsequently, Yaccoby *et al*. [70] have successfully engrafted purified primary cells from MM patients in this system, showing the typical clinical features of MM in the SCID host, such as the appearance of monotypic human Ig in the serum, high blood calcium levels and pathophysiological changes in the implanted human bone. Newly formed blood vessels were also detectable in areas infiltrated by MM cells, demonstrating active angiogenesis. In this model, the activity of the anti-angiogenic agent thalidomide has been demonstrated [71]. The SCID-hu model with primary MM cells growing in the huBMM may better recapitulate the human disease; however, it is hampered by the limited amount of MM cells that can be obtained from the same patient, and therefore only a limited number of mice can be engrafted at the same time to perform treatment studies. 

**Fig. (1):**
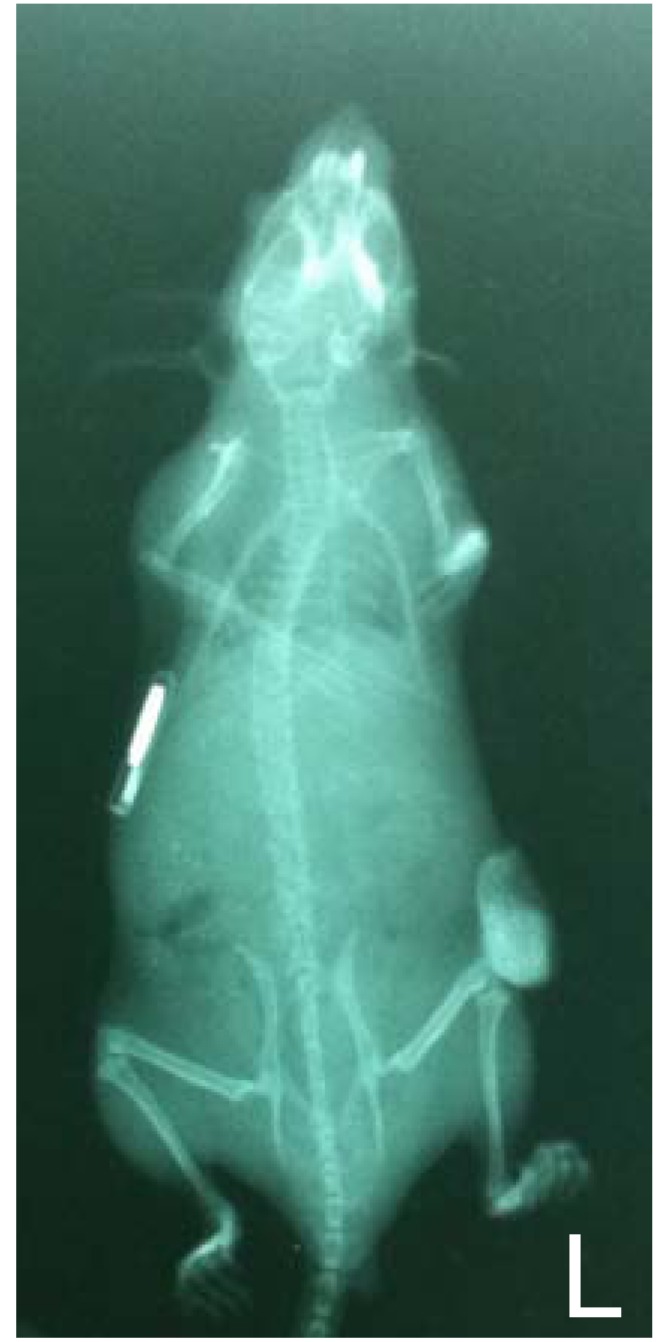
**SCID-hu model**. The radiogram shows a SCID mouse bearing a human fetal bone fragment on the left (L) flank and an electronic identification chip on the right flank.

To overcome these limitations, a variant of the SCID-hu model has been developed by replacing primary MM cells with the IL-6 and BMSC dependent human plasma cell line INA-6 [72]. The cells have been stably transfected with GFP and injected into SCID mice previously implanted with human fetal bone chips (Fig. **1**) Serum levels of soluble huIL-6R, released by INA-6 cells, and external real-time fluorescence imaging of the mice were used as sensitive indicators of tumor growth [73]. Growth of INA-6 cells in the BM was confirmed by histological examination of the bone chips. The INA-6 SCID-hu model is a reliable and manageable system for first-step screening of investigational new compounds. Importantly, *in vivo* growth of the cells is regulated by IL-6/gp130 cytokines, a disease-relevant phenotype, making this model particularly suitable to evaluate cytokine or cytokine receptor antibodies and small-molecule signaling inhibitors such as the IL-6R super-antagonist SANT7 [74], anti-IL-6 antibodies [75], or JAK kinase inhibitors [76, 77]. The INA-6 SCID-hu model has also allowed to demonstrate the anti-MM activity of an anti-BAFF mAb [53], the anti-inflammatory drug Atiprimod [78], the anti-DKK1 mAb BHQ880 [79], the CD138 targeting immunoconjugate B-B4-DM1 [80], the IkappaB kinase beta inhibitor MLN120B [81], and a telomerase inhibitor [82]. However, even if the SCID-hu MM model remains a reliable model to recapitulate the human disease in SCID mice and provides an important preclinical platform for *in vivo* investigation, there are some limitations in the use of this system, in particular (*i*) the restricted availability of human fetal bone chips, (*ii*) the allogeneic nature of the fetal BM *milieu* *versus* MM cells, and (*iii*) the biological heterogeneity of implanted human bone chips, collected from different individuals at different gestational age. 

## THE SCID-rab MODEL

The SCID-rab model has been recently developed in the aim to overcome the limitation of the use of fetal bones in the SCID-hu model [83]. Specifically, the use of an embryonic bone has raised both practical as well as scientific concerns with regard of growing myeloma cells in a stem cell-enriched huBMM. In this model, the human fetal bone has been replaced by a rabbit bone since rabbits are phylogenetically closer to primates than rodents are. The microenvironment in the form of a rabbit bone has demonstrated the ability to support sustained growth of primary human MM cells from the majority of patients. Similar to the SCID-hu model, primary MM cells engraft in the rabbit bone, produce monoclonal immunoglobulin and lead to typical disease manifestations, such as induction of severe lytic bone disease and angiogenesis. Again, similar to the SCID-hu model, tumor cells disseminate only from one rabbit bone to another, demonstrating the ability of the rabbit bone microenvironment to attract MM cells and sustain their growth. Moreover, in this model MM growth was associated with increased numbers of osteoclasts and tumor-associated microvessels. 

Compared to the SCID-hu model, the SCID-rab model has some potential advantages including a more appropriate environment for the study of MM-associated bone disease. The human fetal BM in fact contains a high pool of osteoblast precursors that may not resemble the BM of elderly myeloma patients. In addition, human cells (malignant and nonmalignant) can be easily identified based on species specificity. However, an important disadvantage of the model is the obvious biological differences between the rabbit bone and the human adult bone and the questionable reliability of the biologic significance of cell to cell interactions in a chimeric system. 

## THE LAGlambda-1 MODEL

The LAGlambda-1 model has been recently developed as an approach to reproduce human MM *in vivo* for rapid evaluation of new therapies [84]. Fresh whole core BM biopsies obtained from 33 MM patients were engrafted into the hind limb muscle of SCID mice. In most mice human Ig was detected and some animals developed tumors which were transplanted by intramuscular passages. One of them, an IgG-lambda producing tumor, known as LAGλ (Los Angeles IgG light chain)-1, was selected and underwent further continuous passages in the murine muscle, subcutaneously or by intravenous administration. SCID mice injected with LAGλ cells develop clinical manifestations of human MM including increasing levels of paraprotein, BM plasmacytosis and bone lesions. Moreover, the LAGλ model reproduces a chemoresistant disease and was successfully evaluated for response to established anti-MM agents. 

Overall, the LAGλ model provides some advantages over conventional models for the study of novel drugs in the context of refractory disease. The expansion of tumor cells by several passages allows to generate a large cohort of SCID mice bearing tumors for treatment studies, however, in a nonorthotopic environment. Furthermore, the model does not warrant the recapitulation of the heterogeneity of the disease. One disadvantage may be that the tumor cells require the *in vivo* environment to grow and undergo apoptosis in ex-vivo cultures. 

## THE TURKEY EMBRYOS XENOGRAFT MODEL 

A turkey embryos xenograft model has been recently described as a rapid low-cost *in vivo* system for engraftment of MM cells in pre-immune turkey [85]. In this model, cell lines and malignant plasma cells in primary culture from MM patient cells were injected intravenously into embryonic veins. The engraftment was achieved after 1 week in all embryos injected with cell lines and in 50% of those injected with patient cells. The treatment with bortezomib or lenalinomide 48h after cell injection at therapeutic doses reduced MM engraftment, suggesting the suitability of this rapid system for the study of novel therapeutics *in vivo*. In the report, the authors suggest that this model may facilitate faster *in-vivo* screening of anti-MM agents, thereby reducing drug development time and cost, providing a venue for personalized treatment. However, there are some limitations in the use of this model: i) the number of MM cells is limited in the BM samples harvested from an individual patient, thus limiting the number of embryos that can be injected with cells from a single patient; ii) the reduced time allowed for engraftment in embryos limits the successful use of all sample due to the high variability in the time to engraftment of MM samples; and iii) there are obvious differences in the biology of a turkey microenvironment as compared to a human *milieu* and again there is a major concern on the biological relevance on cell interactions in a chimeric system.

## THE SCID-*synth-hu* MODEL

**Fig. (2):**
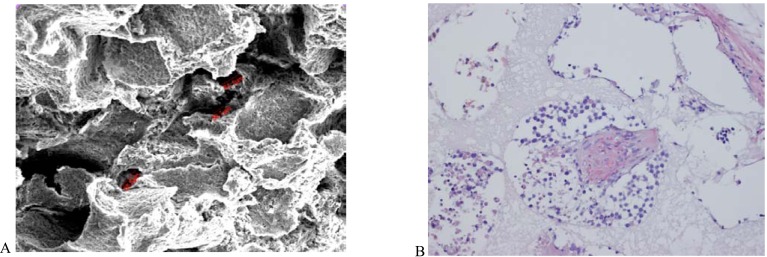
**SCID-synth-hu model**. **A**: Microarchitecture of a scaffold section showing interconnected small pores at scanning electron microscopy (SEM) analysis. **B**: Histological section of a biosynthetic scaffold retrieved from a SCID mouse. The scaffold was first implanted with human bone marrow stromal cells from MM patients and then engrafted with human MM cells.

In the aim to overcome the limitations of the SCID-hu system, a new model, the SCID-*synth-hu*, has been recently developed [86, 87]. This model is based on the implantation into a SCID mouse of a three-dimensional (3D) bone-like poly-ε-caprolactone polymeric scaffold (PCLS) (Fig. **2A**). This synthetic recipient accurately reproduces the micro-architecture of a normal human femur adult bone. It allows efficient coating of the 3D scaffold internal surface with human BMSCs yielding an environment that successfully supports the engraftment of human primary MM cells (Fig. **2B**). This model overcomes one important limitation associated with the SCID-hu model: the allogenic nature of the environment. In fact, injection within the biosynthetic scaffold of the whole unselected cell population from BM aspirates, containing both primary CD138^+^ MM cells and their autologous BMSCs, resulted in a successful engraftment of all patient samples. The possibility to achieve an engraftment of primary MM cells within their micro-environment represents an important advancement in the availability of powerful *in vivo* platforms for the study of MM. Importantly, in their report, the authors assessed the suitability of the system for preclinical evaluation of anti-MM agents. The treatment of MM-bearing SCID-synth-hu mice with bortezomib plus dexamethasone dramatically inhibited MM cell growth *in vivo*, demostrating that this model is suitable for large-scale *in vivo* preclinical screening of novel drugs. An additional value of the model is the detection of vasculogenetic events within biosynthetic scaffolds together with the presence of a neosynthesized extracellular matrix. As compared with the SCID-hu model, the SCID-synth-hu offers a number of potential advantages including the unlimited availability of PCLSs and the possibility to dissect biological events within the huBMM by the use of genetically manipulated cell populations. Most importantly, the SCID-synth-hu allows engraftment of primary MM cells in a non-fetal adult autologous huBMM, therefore representing a unique tool for translational research in MM.

## SUMMARY

This review highlights strengths and weaknesses of the most commonly used and newly developed innovative preclinical *in vivo* models of MM (Table **1**). Essentially, they involve transplantation and engraftment of established murine or human cell lines in syngenic or immuno-compromised mice, respectively. Xenograft models are useful tools for initial screening of innovative drugs particularly in the absence of pharmacological information but should not be considered as ideal models for anti-MM drug development. In fact, the non-human host stroma raises concerns on the relation to the pathophysiological situation in patients. Mouse xenograft models can therefore mostly serve as useful filters for defining the anti-tumor activity and the pharmacodynamic properties of a potential anti-MM drug. On the other hand, transgenic mouse models of MM allow definition of biological steps in the pathogenesis of the disease and may be useful to test drugs that specifically target the genetic lesion introduced to cause the disease, but are inadequate for large scale screening of investigational drugs.

Innovative models based on the engraftment of human primary MM cells within a huBMM obviously overcome most important weaknesses of conventional xenografts or genetically modified mice. They are useful to investigate disease pathophysiology and for preclinical evaluation of investigational drugs targeting not only the tumor compartment but also the huBMM. However, even if the SCID-hu model represent the most powerful systems to validate innovative drugs in MM, significant differences may exist between the human fetal environment as compared to the elderly patient bone. In this light, the recently developed SCID-synth-hu model, may offer the possibility to overcome this important limitation [86, 87]. In fact, this model recapitulates the growth of primary MM cells within a 3D biosynthetic recipient coated with allogeneic or autologous adult human BM stroma. By using genetically engineered stromal cells or manipulating other components of the huBMM, this system allows the dissection of biological events occurring within the huBMM, that may play a role in the pathophysiology of the disease and in the molecular mechanisms of investigational drugs. An additional important point is the plasticity of the model that allows to investigate molecular variants of the human disease by creating personalized models for each patient. This may be relevant in the light of the fact that MM drug development cannot rely on the “one-fits-all” concept taking into account the molecular heterogeneity of the disease. The rising interest for novel therapeutics based on the interference, for instance, with the miRNA network raises additional needs. Such networks, more than conventional targets, are highly dependent on tumour cell growth conditions and specific interactions with the huBMM. Therefore, availability of preclinical models able to recapitulate an autologous tumour-stroma interaction might be a relevant tool for *in vivo* studies in this specific setting. However, more efforts are needed in this field to further improve preclinical platforms and to allow their routinely use for the evaluation of new anti-MM agents. 

## Figures and Tables

**Table 1.** Summary of biological features and potential applications of MM *in vivo* models.

Model	Human MM	Murine MM	Human *milieu*	Animal *milieu*	Suitability for Drug Screening	Suitability for Immunotherapeutic Studies
5T series		✓		✓	✓	✓
Engineered		✓		✓	Restricted*	✓
Conventional xenografts	✓			✓	✓	
SCID-*hu*	✓		✓		✓	
SCID-*rab*	✓			✓	✓	
LAGlambda-1	✓			✓	✓	
Turkey embryos xenograft	✓			✓	✓	
SCID-*synth-hu*	✓		✓		✓	

**Abbreviations:** human *milieu* = human bone marrow microenvironment; animal *milieu* = orthotopic or nonhorthotopic animal microenvironment.

*: mostly for agents targeting the specific genetic lesion introduced in the animal.

## ABBREVIATIONS

BM: = Bone marrow
BMM: = Bone marrow microenvironment
BMSCs: = Bone marrow stromal cells
huBMM: = Human bone marrow microenvironment
LAGλ: = Los Angeles IgG light chain
MM: = Multiple myeloma
NOD: = Non–obese diabetic
SCID: = Severe combined immunodeficient
SCID-hu: = Severe combined immunodeficient-human
SCID-synth-hu: = Severe combined immunodeficient-biosynthetic-human
